# The Association of Plasma Osmolarity With No-Reflow in Patients With ST-Segment Elevation Myocardial Infarction: A Retrospective Cohort Study

**DOI:** 10.7759/cureus.86521

**Published:** 2025-06-22

**Authors:** Faraz Ahmad, Ahmad Usman, Aneela Afreen, Iffat Aqeel, Tayyab Farooq, Atif Nadeem, Rana Hanan, Ali Raza

**Affiliations:** 1 Cardiology, Shalamar Hospital, Lahore, PAK; 2 Cardiology, Integrated Medical Care Hospital, Lahore, PAK; 3 Cardiology, Army Cardiac Center, Combined Military Hospital (CMH), Lahore, PAK; 4 Internal Medicine, Shalamar Hospital, Lahore, PAK

**Keywords:** no-reflow, percutaneous coronary intervention, plasma osmolarity, reperfusion, st elevation myocardial infarction

## Abstract

Background

The no-reflow phenomenon is a serious complication that can occur after primary percutaneous coronary intervention (PCI) in patients with ST-segment elevation myocardial infarction (STEMI), leading to poor myocardial reperfusion and adverse outcomes.

Objective

This study aims to evaluate the association between admission plasma osmolarity and the occurrence of the no-reflow phenomenon in patients with STEMI undergoing primary PCI.

Methods

This retrospective cohort study was conducted at Shalamar Hospital, Lahore, Pakistan from February 2022 to February 2025. The study included 486 consecutive patients diagnosed with STEMI who underwent primary PCI. Patients aged ≥ 18 years, confirmed diagnosis of STEMI based on chest pain duration of more than 30 minutes, ST-segment elevation of ≥1 mm in at least two contiguous ECG leads, and elevated cardiac biomarkers were included in the study.

Results

In this study of 486 STEMI patients undergoing primary PCI, high plasma osmolarity (>295 mOsm/L) was significantly associated with an increased incidence of no-reflow (31.1%) compared to normal (13.4%) and low osmolarity groups (9.2%). The incidence of no-reflow was significantly higher in the high osmolarity group (31.1%) compared to the normal (13.4%) and low (9.2%) groups (p < 0.001). High plasma osmolarity was independently associated with an increased risk of no-reflow (adjusted odds ratio 2.94; 95% CI: 1.61-5.38; p < 0.001).

Conclusion

It is concluded that elevated plasma osmolarity is an independent predictor of the no-reflow phenomenon in STEMI patients undergoing primary PCI. Routine assessment of plasma osmolarity may offer a simple and effective method for early risk stratification, with potential implications for personalized management strategies to improve reperfusion outcomes.

## Introduction

ST-segment elevation myocardial infarction (STEMI) is an urgent medical problem and ranks among the leading reasons for death and lasting disability worldwide. The main focus of how to manage patients involves timely reperfusion, most often through primary percutaneous coronary intervention (PCI) [1]. PCI can restore the blood flow in the artery, but a good number of patients still fail to receive enough blood to their heart muscle at the microvascular stage [2]. There is a link between no-reflow and more severe injury, decreased left ventricular function, increased chances of heart arrhythmias and higher rates of death both in the hospital and after release [3].

No-reflow is caused by several things, including small clots further down the blood vessel, swelling of the inner wall, blockage of capillaries by immune cells and side effects of reopening. The problem appears to involve valves in small cells, not obstruction in the large ones and has been related to several risk factors [4]. Deciding which patients might face no-reflow after a procedure is still a hard question in spite of new treatments and approaches [5]. Currently, using thrombus burden, how long ischemia persists and diseases like diabetes or kidney illness as predictors is helpful, but not enough on its own. It is still necessary to have biomarkers that are dependable, accessible and migrate, so clinicians can determine which patients are most at risk during PCI [6].

A promising yet not widely studied factor is plasma osmolarity, which measures how many sodium, potassium, glucose and urea particles are in the blood. It incorporates different aspects of human function, including hydration, electrolyte state and control of blood glucose [7]. Having either an increase or a decrease in plasma osmolarity may strongly affect the tone of blood vessels, the condition of the endothelium and processes related to inflammation. Hyperosmolar problems may also result in higher oxidative stress, harm to endothelial cells and the activation of pro-inflammatory pathways, all leading to microvascular obstruction. By contrast, hypo-osmolarity can cause fluid to build up in cells, reduce muscle contraction in the heart and further harm tissue suffering from ischemia [8]. Plasma osmolarity has been associated with a greater number of bad heart-related outcomes in critically ill patients [9]. Experts report that too high or too low osmolarity is linked to increased death risk in situations such as heart failure, sepsis and traumatic brain injury [10]. People with acute coronary syndromes who have hyperosmolar blood have been linked to bigger heart attacks and poorer outcomes. At the moment, it is not certain what relationship exists between plasma osmolarity and the no-reflow problem in patients whose STEMI is addressed with PCI [11].

Objective

This study aims to evaluate the association between admission plasma osmolarity and the occurrence of the no-reflow phenomenon in patients with STEMI undergoing primary PCI.

## Materials and methods

Methodology

This retrospective cohort study was conducted at Shalamar Hospital, Lahore, Pakistan from February 2022 to February 2025. The study included 486 consecutive patients diagnosed with STEMI who underwent primary PCI.

Inclusion and exclusion criteria

Patients aged ≥ 18 years, confirmed diagnosis of STEMI based on chest pain duration of more than 30 minutes, ST-segment elevation of ≥1 mm in at least two contiguous ECG leads, and elevated cardiac biomarkers were included in the study. All patients had undergone primary PCI within 12 hours of symptom onset. Patients with a history of coronary artery bypass grafting (CABG), end-stage renal disease requiring dialysis, presence of chronic liver disease, active systemic infection, malignancy, prior thrombolytic therapy for the current event, or missing laboratory data necessary for plasma osmolarity calculation were excluded. Patients with partial data were excluded only if the missing information pertained to key variables required for the primary analysis (e.g., sodium, glucose, blood urea nitrogen (BUN)). For non-critical missing values, complete case analysis was performed, and no data imputation techniques were applied.

Data collection

Clinical and demographic data, including age, sex, cardiovascular risk factors (hypertension, diabetes mellitus, smoking, and dyslipidemia), infarct location, Killip classification, and symptom-to-balloon time, were retrieved from medical records. Laboratory parameters were obtained on admission, prior to PCI. These included serum sodium, glucose, and BUN, which were required to calculate plasma osmolarity. Plasma osmolarity was calculated using the following validated formula:

Plasma Osmolarity (mOsm/L) = (2 × Na⁺) + (Glucose/18) + (BUN/2.8)

Where Na⁺ = serum sodium in mmol/L, Glucose = blood glucose in mg/dL, and BUN = blood urea nitrogen in mg/dL.

All values were expressed in standard clinical units (Na⁺ in mmol/L, glucose and BUN in mg/dL). Based on the calculated osmolarity, patients were stratified into three groups: Low osmolarity: < 285 mOsm/L; Normal osmolarity: 285-295 mOsm/L; and High osmolarity: > 295 mOsm/L.

All procedures were performed by experienced interventional cardiologists using the radial or femoral access. Coronary angiography and PCI were conducted according to standard institutional protocols. Post-procedural angiographic flow was assessed using the Thrombolysis in Myocardial Infarction (TIMI) grading system. The no-reflow phenomenon was defined as final TIMI flow grade < 3 in the absence of angiographic evidence of mechanical obstruction, significant residual stenosis, coronary dissection, or vasospasm. Thrombus burden was assessed angiographically using the modified TIMI thrombus grading system at baseline. Grades 0-5 were assigned based on visual estimation, and assessments were confirmed independently by two interventional cardiologists blinded to clinical outcomes.

Statistical analysis

Data were analyzed using SPSS v26 (IBM Corp., Armonk, NY, USA). Continuous variables were presented as mean ± standard deviation or median with interquartile range (IQR) based on data distribution. Categorical variables were expressed as frequencies and percentages. Comparisons between groups were performed using the chi-square test for categorical variables, and one-way analysis of variance (ANOVA) for continuous variables. To determine independent predictors of the no-reflow phenomenon, univariate analysis was initially conducted, followed by multivariate logistic regression modeling. A two-sided p-value of < 0.05 was considered statistically significant.

## Results

Data were collected from 486 patients. The mean age was slightly higher in the high osmolarity group (62.9 ± 11.5 years) compared to the normal (61.0 ± 11.3 years) and low (60.2 ± 10.9 years) osmolarity groups, although this difference was not statistically significant (p = 0.173). The proportion of male patients was similar across all groups: 68.4% in the low, 70.4% in the normal, and 72.3% in the high osmolarity group (p = 0.428). A significant finding was the higher prevalence of diabetes mellitus in the high osmolarity group (42.6%) compared to the normal (29.6%) and low (24.3%) groups (p = 0.002), suggesting a strong association between hyperosmolarity and metabolic dysregulation. Symptom-to-balloon time also showed a significant difference, being longest in the high osmolarity group (5.8 ± 2.3 hours), followed by the normal (5.1 ± 1.9 hours) and low (4.9 ± 2.0 hours) groups (p = 0.038), indicating delayed intervention among patients with higher osmolarity. Other factors such as hypertension (55.9%, 58.1%, and 60.8%; p = 0.569) and smoking status (40.8%, 41.4%, and 45.3%; p = 0.498) did not differ significantly across the groups (Table 1).

**Table 1:** Baseline Characteristics by Osmolarity Group Data are represented as Mean ± SD or percentages (%). Significance level: p < 0.05.

Characteristic	Low Osmolarity (<285)	Normal Osmolarity (285-295)	High Osmolarity (>295)	p-value
Number of patients	152	186	148	-
Age (years)	60.2 ± 10.9	61.0 ± 11.3	62.9 ± 11.5	0.173
Male (%)	68.4%	70.4%	72.3%	0.428
Diabetes mellitus (%)	24.3%	29.6%	42.6%	0.002
Hypertension (%)	55.9%	58.1%	60.8%	0.569
Smoking (%)	40.8%	41.4%	45.3%	0.498
Symptom-to-balloon time (hours)	4.9 ± 2.0	5.1 ± 1.9	5.8 ± 2.3	0.038

Among patients with low osmolarity (<285 mOsm/L), 14 out of 152 (9.2%) experienced no-reflow. In the normal osmolarity group (285-295 mOsm/L), the incidence increased to 25 out of 186 patients (13.4%). Notably, the highest rate was observed in the high osmolarity group (>295 mOsm/L), where 46 out of 148 patients (31.1%) developed no-reflow (Table 2).

**Table 2:** Incidence of No-Reflow by Osmolarity Group

Osmolarity Group	No-Reflow Cases (n)	Total Patients (n)	No-Reflow Incidence (%)
Low (<285)	14	152	9.2
Normal (285–295)	25	186	13.4
High (>295)	46	148	31.1

High plasma osmolarity (>295 mOsm/L) was strongly associated with an increased risk of no-reflow, with an adjusted odds ratio (OR) of 2.94 (95% CI: 1.61-5.38; p < 0.001). In contrast, low osmolarity (<285 mOsm/L) was not significantly associated with no-reflow (OR: 0.71; 95% CI: 0.36-1.39; p = 0.316). Diabetes mellitus emerged as a significant predictor (OR: 1.89; 95% CI: 1.12-3.21; p = 0.017), along with high thrombus burden (OR: 2.25; 95% CI: 1.32-3.84; p = 0.003). Additionally, a symptom-to-balloon time greater than six hours was associated with an increased likelihood of no-reflow (OR: 1.68; 95% CI: 1.01-2.81; p = 0.046) (Table 3).

**Table 3:** Multivariate Logistic Regression Analysis Odds Ratios (ORs) with 95% Confidence Intervals (CI) are reported. Significance level set at p < 0.05.

Variable	Adjusted OR	95% CI	p-value
High Osmolarity (>295)	2.94	1.61–5.38	<0.001
Low Osmolarity (<285)	0.71	0.36–1.39	0.316
Diabetes Mellitus	1.89	1.12–3.21	0.017
High Thrombus Burden	2.25	1.32–3.84	0.003
Symptom-to-Balloon Time > 6 h	1.68	1.01–2.81	0.046

The mean left ventricular ejection fraction (LVEF) was notably lower in the no-reflow group (38.6 ± 7.9%) versus the no no-reflow group (46.4 ± 6.8%) with a p-value < 0.001, indicating impaired cardiac function. In-hospital mortality was also significantly higher among no-reflow patients (9.4%) compared to those without no-reflow (2.1%) (p = 0.004). Furthermore, arrhythmias occurred more frequently in the no-reflow group (22.4% vs. 9.2%; p = 0.001). Patients with no-reflow experienced a longer hospital stay, averaging 7.6 ± 2.4 days compared to 5.3 ± 1.8 days for those without no-reflow (p < 0.001) (Table 4).

**Table 4:** In-Hospital Outcomes by No-Reflow Status Data shown as mean ± SD or percentages (%). Significance level: p < 0.05. LVEF: left ventricular ejection fraction

Outcome	No-Reflow (n=85)	No No-Reflow (n=401)	p-value
LVEF (%)	38.6 ± 7.9	46.4 ± 6.8	<0.001
In-hospital mortality (%)	9.4%	2.1%	0.004
Arrhythmia (%)	22.4%	9.2%	0.001
Length of hospital stay (days)	7.6 ± 2.4	5.3 ± 1.8	<0.001

Aspirin was administered to all patients (100%) regardless of no-reflow status. Use of P2Y12 inhibitors was slightly lower in the no-reflow group (95.3%) compared to the no no-reflow group (98.0%), though the difference was not statistically significant (p = 0.128). Statin use also showed a modest difference (90.6% vs. 95.3%; p = 0.075). However, significant differences were observed in the use of beta-blockers and angiotensin converting enzyme (ACE) inhibitors/angiotensin receptor blockers (ARBs). Beta-blocker administration was lower in the no-reflow group (72.9%) than in the no no-reflow group (79.8%) (p = 0.042). Similarly, ACE inhibitors or ARBs were used less frequently in patients with no-reflow (67.1%) compared to those without (76.2%) (p = 0.031) (Table 5).

**Table 5:** Medication Use on Admission Data shown as percentages (%). Significance level: p < 0.05. ACE: angiotensin converting enzyme, ARBs: angiotensin receptor blockers

Medication	Total Cohort (%)	No-Reflow (%)	No No-Reflow (%)	p-value
Aspirin	100%	100%	100%	-
P2Y12 Inhibitor	97.5%	95.3%	98.0%	0.128
Statins	94.2%	90.6%	95.3%	0.075
Beta-blockers	78.3%	72.9%	79.8%	0.042
ACE Inhibitors/ARBs	74.5%	67.1%	76.2%	0.031

## Discussion

The goal of the study was to discover any link between abnormal plasma osmolarity and the no-reflow problem encountered during primary PCI for STEMI. The results revealed that having above-normal plasma osmolarity is a strong indicator for no-reflow and that plasma osmolarity could independently reveal blockages in the microvasculature during an acute heart attack [12]. No-reflow occurred in 31.1% of those with high plasma osmolarity, compared to only 9.2% in the low plasma osmolarity group and 13.4% in the normal group. Multivariate analysis found that higher plasma osmolarity raises the chances of no-reflow by up to 2.94 times. After taking into account diabetes, the number of clots and extended time before blood flow was restored, these findings showed a continued significant correlation between MI and inflammatory cells [13,14].

This relationship could be caused by a variety of pathophysiological factors. An increased plasm osmolarity points to conditions such as hypernatremia, hyperglycemia or greater urea, each of which is linked to problems like decreased endothelial function, increased oxidative stress and inflammation [15]. Impairment in these processes tends to raise the risk of myocardial injury after blood flow is restored. High concentrations in blood fluids may increase the blood’s viscosity and alter shear stress which makes microvascular blockages more severe and leads to the no-reflow pattern. No statistically significant relationship was found between plasma osmolarity and no-reflow in this group [16]. Despite hypotonicity being able to promote edema and swelling, the results here suggest that acutely, the more significant change is hyperosmolarity. Research in other critical care populations shows that increased blood osmolarity is tied to poor results such as higher death rates and problems with organs [17].

These results have important clinical effects. Routinely measured plasma osmolarity would make it easy to include in models used to determine the risk for STEMI patients. Identifying those at high risk for microvascular injury through osmolarity could help doctors use early measures to address the problem [18]. The treatments might consist of large fluid intake, managing blood glucose, antioxidant medicines or drugs that help with body fluids. The results also support what we already knew about no-reflow such as diabetes, extra time between symptom onset and treatment and a large amount of blood clots [19]. Patients experiencing no-reflow had a poorer clinical status, seen in reduced left ventricular function, more arrhythmias, increased length of time in the hospital and an increased chance of dying in the hospital. Based on these results, making early predictions and preventing no-reflow can improve the likely outcome of patients [20].

This study has several limitations. It was conducted retrospectively at a single center, which may limit the generalizability of the findings. Plasma osmolarity was calculated using a validated formula rather than directly measured, as direct assessment is impractical in routine clinical settings. Patient hydration status at presentation and the influence of concomitant medications were not controlled, which may have affected osmolarity values. The absence of subgroup or sensitivity analyses limits the understanding of whether this association is consistent across clinical subpopulations. Additionally, while patients with missing key data were excluded and complete case analysis was used, no imputation techniques were applied. Thrombus burden was assessed by two blinded cardiologists using the modified TIMI grading system, but inter-rater reliability was not formally evaluated. Variability in clinical documentation may have introduced minor inconsistencies. The angiographic assessment of no-reflow was performed by experienced interventional cardiologists, but blinding was not enforced, introducing the potential for observer bias. Additionally, key variables such as symptom-to-balloon time, myocardial blush grade, and exact thrombus burden were not included in the multivariate analysis, raising the possibility of unmeasured confounding. Patients with missing osmolarity data were excluded, and the potential selection bias was not quantified. Finally, the study focused solely on angiographic outcomes, without evaluating long-term clinical endpoints such as mortality or rehospitalization. Further prospective, multicenter studies are warranted to confirm these findings and explore whether correcting osmolarity can reduce no-reflow incidence and improve clinical outcomes.

## Conclusions

It is concluded that elevated plasma osmolarity is significantly associated with the occurrence of the no-reflow phenomenon in patients presenting with ST-segment elevation myocardial infarction undergoing primary percutaneous coronary intervention. Patients with higher osmolarity levels demonstrated a markedly increased risk of impaired myocardial perfusion, independent of other established clinical and procedural risk factors. Given that plasma osmolarity is easily calculated from routine laboratory parameters, it may serve as a practical and cost-effective tool for early risk stratification in acute myocardial infarction. However, due to the retrospective nature of the study and the possibility of residual confounding, these findings should be viewed as exploratory and hypothesis-generating. Importantly, the results pertain solely to angiographic no-reflow and do not imply an association with long-term clinical outcomes. Further prospective studies, including subgroup analyses and external validation, are needed to confirm the association and clarify its clinical applicability.
